# Discussing global warming leads to greater acceptance of climate science

**DOI:** 10.1073/pnas.1906589116

**Published:** 2019-07-08

**Authors:** Matthew H. Goldberg, Sander van der Linden, Edward Maibach, Anthony Leiserowitz

**Affiliations:** ^a^Yale School of Forestry and Environmental Studies, Yale University, New Haven, CT 06511;; ^b^Department of Psychology, University of Cambridge, CB2 3EB Cambridge, United Kingdom;; ^c^Department of Communication, George Mason University, Fairfax, VA 22030

**Keywords:** climate change, discussion, scientific consensus, self-persuasion, climate change communication

## Abstract

Climate change is an urgent global issue, with demands for personal, collective, and governmental action. Although a large body of research has investigated the influence of communication on public engagement with climate change, few studies have investigated the role of interpersonal discussion. Here we use panel data with 2 time points to investigate the role of climate conversations in shaping beliefs and feelings about global warming. We find evidence of reciprocal causality. That is, discussing global warming with friends and family leads people to learn influential facts, such as the scientific consensus that human-caused global warming is happening. In turn, stronger perceptions of scientific agreement increase beliefs that climate change is happening and human-caused, as well as worry about climate change. When assessing the reverse causal direction, we find that knowing the scientific consensus further leads to increases in global warming discussion. These findings suggest that climate conversations with friends and family enter people into a proclimate social feedback loop.

Climate change is a global issue, requiring personal, collective, and governmental action (1). Most prior research on how to motivate action has focused on understanding individual differences, for example, considering the role of education, religion, and ideology in driving climate change belief polarization (2–4). Other research has focused on how top-down communications, for example, from scientists, influence public beliefs (5). Importantly, however, little research has focused on understanding how interpersonal conversations shape beliefs and worry about climate change. This is surprising, considering the importance of the messenger in communication (2, 3), and the fact that friends and family are one of the most trusted sources of climate change information (6). Although people seldom discuss climate change with friends and family (7), discussion with others in one’s close social network can be an important route by which people may learn key facts about an issue, such as the scientific consensus on climate change. As such, it is important to investigate the influence of people’s climate conversations with their own friends and family.

Moreover, while a rapidly growing body of research has examined the influence of messages on climate change public engagement, few studies have investigated the role of interpersonal discussion (but see ref. 8). In this study, we use nationally representative panel data to examine the influence of discussion about climate change on public climate change beliefs over time.

One fact that influences people’s climate beliefs is the degree to which people perceive a scientific consensus about human-caused climate change (9, 10). Do people learn about the scientific consensus on climate change through discussion with family and friends? If so, does this have downstream effects on beliefs and worry about climate change? Answers to these questions may suggest that encouraging people engaged with climate change to discuss it with their less-engaged friends and family members could be an effective strategy to increase public engagement through social network activation.

Next, we investigate the possibility of reciprocal causation. That is, are people who perceive higher scientific agreement more likely to discuss climate change with friends and family, which reinforces their own beliefs and worry about climate change?

## Study Overview

A nationally representative probability sample of US adults (*n* = 1,263) was surveyed at 2 time points about 7 mo apart. We used the SEM module in STATA (version 15) to conduct a cross-lagged panel analysis investigating 1) changes in perceptions of scientific consensus as a result of discussion with family and friends, 2) changes in climate change discussion as a result of perceptions of the scientific consensus, and 3) the indirect effects of discussion and consensus beliefs on cognitive and affective judgments about climate change.

## Results

**Fig. 1.**
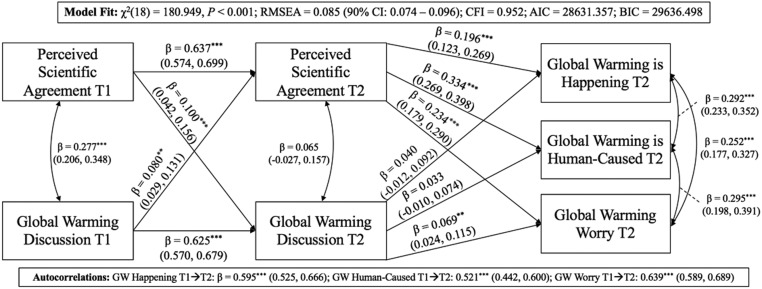
Coefficients are standardized. Ninety-five percent CIs were derived using 1,000 bootstrap samples (*n* = 1,263). Global warming (GW) beliefs (happening and human-caused) and worry autocorrelations are depicted below the figure to preserve legibility. **P < 0.01; ***P < 0.001. T1, time 1; T2, time 2; RMSEA, root mean square error of approximation; CFI, comparative fit index; AIC, Akaike information criterion; BIC, Bayesian information criterion.

Results from the cross-lagged panel analysis and downstream effects on global warming beliefs and worry are shown in Fig. 1. The results provide evidence for reciprocal causality. That is, discussion of global warming at time 1 led to increased perceptions of scientific agreement at time 2 (*β* = 0.080, 95% CI [0.029, 0.131]), and, equally, perceptions of scientific agreement at time 1 led to increases in global warming discussion at time 2 (*β* = 0.100, 95% CI [0.042, 0.156]). These findings demonstrate a change in each variable at time 2 because the model controls for scores of the same variables at time 1 (see ref. 9).

Increases in perceptions of scientific agreement over time, in turn, led to significant increases in the belief that global warming is happening and human-caused, as well as worry about global warming. Increases in discussion over time did not lead to downstream increases in the belief that global warming is happening or human-caused, but did lead to increases in worry (Fig. 1).

The indirect effect of discussion via an increase in perceptions of scientific agreement was significant for the belief that global warming is happening (*β* = 0.015, 95% CI [0.013, 0.016]), the belief that global warming is human-caused (*β* = 0.025, 95% CI [0.021, 0.030]), and worry (*β* = 0.018, 95% CI [0.013, 0.023]). Likewise, the indirect effect of perceptions of scientific agreement via an increase in global warming discussion was significant for the belief that global warming is happening (*β* = 0.004, 95% CI [0.003, 0.004]), the belief that global warming is human-caused (*β* = 0.003, 95% CI [0.002, 0.004]), and worry (*β* = 0.007, 95% CI [0.001, 0.012]). Compared with the indirect effects of perceived scientific agreement, the indirect effects of discussion were significantly stronger for the belief that global warming is happening (*Z* = 13.879, *P* < 0.001), the belief that global warming is human-caused (*Z* = 8.787, *P* < 0.001), and worry (*Z* = 2.992, *P* = 0.003).

## Discussion

Despite the influence of social networks on individual beliefs and behaviors, the role of climate change discussions with friends and family has received little research attention. Here we find that discussion can generate a feedback loop where people who discuss global warming become more likely to learn influential facts such as the scientific consensus that humans are causing global warming, which encourages further discussion. Importantly, indirect effects of discussion were significantly stronger than those of the scientific consensus, suggesting that encouraging people to discuss global warming with their friends and family may be a productive way to initiate the social feedback loop, but that the actual content of the discussion itself (e.g., scientific agreement) plays a key role in changing relevant beliefs.

The role of global warming discussion among one’s own social network may be especially important given the powerful influence of messengers on message effects (2, 3). For example, when the message comes from close friends and family, people less engaged with the issue may be more receptive than when an identical message is communicated by someone not part of their close social network.

Moreover, the politicization of climate science is likely exacerbated by the increasing fragmentation of media consumption (11). Discussion with others in one’s close social network, on the other hand, appears to be an important route by which people learn key facts about an issue, such as the scientific consensus. Without discussing global warming, people may never learn important facts about climate change, or that close friends and family care about the issue. Our findings show that, through discussion, people can engage their friends and family in a positive feedback loop that encourages deeper engagement with the issue of climate change.

## Materials and Methods

### Participants.

In the first wave of data collection (fielded 27 February to 10 March 2015), a nationally representative probability sample of respondents was recruited through Growth from Knowledge’s Knowledge Panel (*n* = 1,263). Respondents were contacted again approximately 7 mo later (completion rate = 72%; *n* = 905). To avoid potential biases resulting from missing data, missing values were estimated using full information maximum likelihood (12).

### Procedure and Materials.

Respondents were recruited as part of the Climate Change in the American Mind project to participate in a survey on global warming beliefs, attitudes, and policy preferences. To measure global warming discussion, we asked, “How often do you discuss global warming with your family and friends?” (1 = *Never*, 4 = *Often*). Respondents reported their estimates of scientific agreement by answering, “To the best of your knowledge, what percentage of climate scientists think that human-caused global warming is happening?” (0–100%). The question gauging whether respondents believe global warming is happening was “...Do you believe that global warming is or is not happening?” (1 = *No*, 2 = *Don’t Know*, 3 = *Yes*). For human-causation, respondents answered, “Assuming global warming is happening, do you think it is...” (1 = *None of the above because global warming is not happening*, 2 = *Caused mostly by natural changes in the environment*, 3 = *Caused mostly by human activities*). To measure worry about global warming, respondents were asked, “How worried are you about global warming?” (1 = *Not at all worried*, 4 = *Very worried*). Questions were identical at both time points.
